# COVID-19: a challenge and an opportunity

**DOI:** 10.1038/s41431-022-01142-6

**Published:** 2022-07-15

**Authors:** Alessandra Renieri

**Affiliations:** 1 Med Biotech Hub and Competence Center, Department of Medical Biotechnologies, University of Siena, Siena, Italy; 2 Medical Genetics, University of Siena, Siena, Italy; 3 Genetica Medica, Azienda Ospedaliero-Universitaria Senese, Siena, Italy

## Abstract

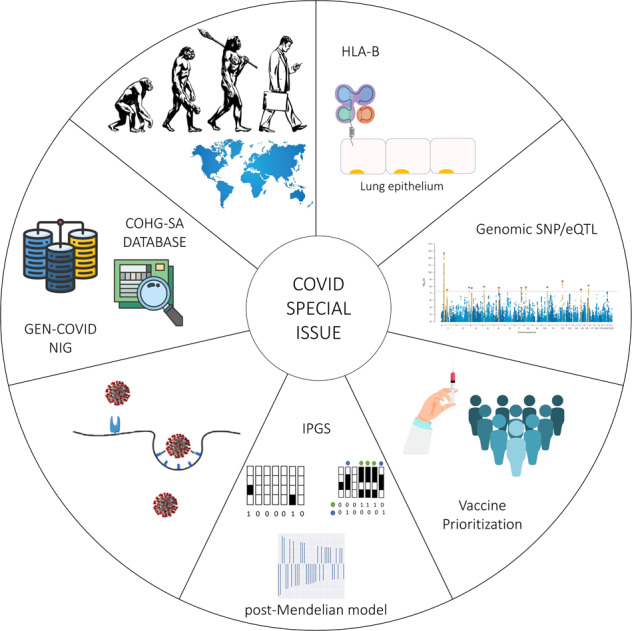

The hereditary laws have been postulated 157 years ago by the mathematician and biologist Gregor Mendel. It is pretty astonishing that these laws are still used today in the current clinical practice. In the Mendelian disorders, also known as monogenic or rare diseases, these laws are used in a number of clinical actions from prenatal, to presymptomatic, and preimplantation diagnosis to target treatment by both traditional and advanced therapy, such as gene therapy.

On the other hand, no substantial advances have been made in mathematical rules useful in clinical practice for complex disorders, in which more than one gene is involved together with environmental factors. In the dualism between genetic Variability (gV) and environmental Variability (eV) the environmental factors have been often elusive. For example, in diabetes putative viral infections have been invoked but never fully defined. The same is true for multiple sclerosis, for which, only very recently a subtype of EBV virus has been likely identified as the key environmental factor.

COVID-19 is the first disease in which environmental factors are well defined, misurabile and weighable. Heritability (h2) as calculated by gV/gV+aV is very high in COVID-19, similarly to psychiatric disorders [1]. While media are taking care mainly of the viral genome, the host genome is the real discriminant between severe COVID-19 and asymptomatic SARS-CoV-2 infection. Therefore, while COVID-19 is representing a challenge for the health systems and society, it is the first real opportunity to move forward on understanding mechanisms and new rules useful in clinical practice of complex disorders, as described in the papers of this issue [1–9].

This special issue wants to summarize for the EJHG readers the key steps toward these achievements. One can start reading the manuscript of Jemison et al which focuses on the SARS-CoV-2 virus’ mechanism of infection and its change of the intracellular environment [2] and the manuscript by Redin et al focusing on the host immunological determinant of SARS-CoV-2 infection [3].

To better understand the pathogenic mechanism of SARS-COV-2 infection and COVID-19 disease, one cannot ignore the HLA system [4]. Zhang Y et al. show an increased allelic expression pattern with overexpression of HLA-B gene in human lung epithelial cells infected by SARS-CoV-2. They also demonstrate that the antiviral cytokine IFN-β contributes to allelic expression of the HLA-B gene in lung cells [4].

Several useful tools are also presented in this issue [5] and other issues of this journal [6] for either updating the readers on loci/genes associated with COVID-19 [5] or genetic data/biological samples availability for running new models [6].

Bruce J at al. explore how host genetics may impact decisions about vaccine administration. As host genetic factors influence vulnerability, as well as resistance to infection, they evaluate the prioritisation of genetic vulnerability in vaccination schemes, and the potential for ethical de-prioritisation for resistors [7]. Another unexplored area about vaccines is related to the so-called “more common” rare disease with impact on lung function such as α-1 antitrypsin (A1AT) deficiency (AATD) [8]. On one hand, AATD patients have not been included in the COVID-19 vaccine clinical trials and vaccine efficacy is unknown. On the other hand, A1AT could be a promising therapeutic option for patients with COVID-19 [8].

A different perspective was employed by Kerner and Quintana-Murci [9]. They focused on past selection events relevant for the history of our species. Admixture with Neanderthals was crucial for modern human adaptation to the threats of pathogens during evolution. They show how ancient genetic adaptation contributes to the observed population differences in the susceptibility to SARS-CoV-2 infection and the severity of COVID-19.

Finally, Zguro et al. focus on new methods for understanding the host genetics determinants of COVID-19 [1]. A new method named “post-Mendelian model” has been developed combining for the first time both common and rare genetic variability and using a gene-based machine learning approach with the support of GEN-COVID (https://sites.google.com/dbm.unisi.it/gen-covid). Data are shared through NIG - Network of Italian Genomes (http://nigdb.cineca.it/). Therefore, we are looking beyond Mendelian rules, making a real step forward in understanding and predicting complex disorders.
